# The Visual Callosal Connection: A Connection Like Any Other?

**DOI:** 10.1155/2013/397176

**Published:** 2013-03-24

**Authors:** Kerstin E. Schmidt

**Affiliations:** ^1^Brain Institute, University of Rio Grande do Norte, Av. Nascimento de Castro 2155, 59056-450 Natal, RN, Brazil; ^2^Max-Planck-Institute for Brain Research, Deutschordenstrasse 46, 60528 Frankfurt, Germany

## Abstract

Recent work about the role of visual callosal connections in ferrets and cats is reviewed, and morphological and functional homologies between the lateral intrinsic and callosal network in early visual areas are discussed. Both networks selectively link distributed neuronal groups with similar response properties, and the actions exerted by callosal input reflect the functional topography of those networks. This supports the notion that callosal connections perpetuate the function of the lateral intrahemispheric circuit onto the other hemisphere. Reversible deactivation studies indicate that the main action of visual callosal input is a multiplicative shift of responses rather than a changing response selectivity. Both the gain of that action and its excitatory-inhibitory balance seem to be dynamically adapted to the feedforward drive by the visual stimulus onto primary visual cortex. Taken together anatomical and functional evidence from corticocortical and lateral circuits further leads to the conclusion that visual callosal connections share more features with lateral intrahemispheric connections on the same hierarchical level and less with feedback connections. I propose that experimental results about the callosal circuit in early visual areas can be interpreted with respect to lateral connectivity in general.

## 1. Introduction

In the mammalian brain, connections between homologous areas extend through the corpus callosum and link the feature spaces represented in the two hemispheres and separated at the body's midline. Many functions have been attributed to callosal connections and a great anatomical variety in degree of myelination and fibre diameter has been described as being compatible with direct or indirect excitatory and also inhibitory function [1]. Most likely, the corpus callosum is a collection of different pathways whose function is critically linked to the species and the cortical area that is interconnected. 

Accordingly, different types of callosal actions can be observed. Those include actions which based on an anatomical asymmetry contribute to the functional asymmetry of some cognitive systems in higher mammals, others which result from the specific position of the connections within the brain's topography, and finally those which simply extend the intrinsic network of intracortical short- and long-range connections onto the contralateral hemisphere in order to ensure functional integrity over the midline [2, 3].

The first group certainly includes actions contributing to the lateralization of higher cognitive functions. Here, one hemisphere might inhibit the other hemisphere in order for one function to predominate and this creates hemispheric dominance for the respective system.

Connections in the motor system responsible for bimanual coordination may mediate the second group of actions. Possibly also to this group belong callosal connections in the visual system of front-eyed animals, which due to their localization evidently act in that part of the visual field where stereoscopic function is expressed. 

The last group might dominate in primary sensory cortices where the sensory periphery is separated into the two hemispheres at the body's midline and has to be united via the callosal connection. Based on anatomical and functional evidence there is reason to believe that these connections perpetuate the intrahemispheric function of the lateral and intrinsic network into the other hemisphere. As such intrinsic and callosal connections could be parts of the same circuit at the same level of cortical hierarchy.

In this paper, recent work about callosal connections between homotopic areas of the primary visual cortices (areas 17 an 18) and their contribution to visual processing in binocular mammals will be embedded into the background of previous research. Arguments for a specific callosal versus perpetuation of intrahemispheric functions will be discussed. This work has been obtained in carnivores, that is, ferrets and cats, using cortical deactivation. Further, most of the mentioned previous work using similar approaches also stems from cats unless stated otherwise.

## 2. Morphology Concerning Visual Callosal Connections

Virtually all retinotopically defined areas in the two hemispheres are densely interconnected by callosal connections [4, 5]. In the primary visual cortices, their density is greatest at the border between areas 17 and 18 where the vertical meridian of the visual field is represented [6–8]. Thus, early on they were suspected to simply unify the two half representations of the visual field in the two hemispheres [7, 9]. In accordance with this, the split-chiasm preparation confirmed that indirect input through the corpus callosum matches the direct and ipsilateral responses in orientation preference and receptive field position [3, 10]. 

In carnivores' early visual areas, visual callosal connections between the two hemispheres clearly accumulate at the areal borders (for review see [11]) but the higher the interconnected areas are situated in the cortical hierarchy the less confined callosal connections seem to be and also the less homotopic (cat: [4, 12]; ferret: [13]). This might be expected from the increasing receptive field sizes.

More detailed studies of the connectivity at the 17/18 border in cats revealed nonhomotopic connectivity patterns already in primary visual cortex. Neurons in areas 17 and 18 seem to project to the 17/18-transition zone on the contralateral hemisphere, whereas projections originating from the 17/18-transition zone terminate preferentially in area 17 or 18 on the other side [14–16]. 

The retinotopic relationship though seems to be always maintained as clearly acallosal regions have been associated with the visual periphery and callosal zones with central visual field representations in both cats [12] and ferrets [17] and also tree shrew [18]. This was also confirmed on the single cell level [19].

In cats, callosal axons originate from and terminate on similar classes of cells in supragranular and to a lesser extent in infragranular layers in cats (for review see [11]). The majority of fibres stem from pyramidal and spiny stellate cells in layer III, superficial layer IV, and layer VI [4, 20–22]. This has been largely confirmed by complete reconstructions of single axons demonstrating in detail that most of the synaptic boutons are situated in layer III, one-third in layer IV, and only a minority in infragranular layers [19]. In contrast, visual callosal connections dominate in infragranular layers in ferrets [13], as also in rodents [23]. 

In several mammals, a part of the ipsilateral visual field is represented bilaterally in areas 17 and 18 [24–26]. In the cat, the ipsilateral representation in the contralateral hemisphere increases from 4° in central parts to 23° towards upper and lower elevations [12, 18, 27] accompanying the visual field magnification and also the extent of the callosally projecting zone. In more lateral-eyed animals like ferrets, tree shrews, or sheep, the zone of overlap is even greater but seems to be confined to area 17 [28–31] where columns of the ipsilateral eye dominate. Information about the ipsilateral field is likely conveyed by ganglion cells in the temporal retina that project to the medial interlaminar nucleus of the lateral geniculate nucleus [32–34] which then projects to the 17/18 border [27]. Even though callosal connections could possibly extend over a larger zone than the bilaterally represented stripe, it would be difficult to clarify if and where in the visual field callosal input provides information, which cannot be provided by the feedforward geniculocortical loop. More likely, this composite organization strengthens the hypothesis that callosal connections in the early visual areas perpetuate the intrahemispheric lateral network and- under normal circumstances-serve rather modulatory than feedforward driving functions.

However, when the geniculocortical input is taken away by the split-chiasm preparation, callosal connections are able to directly—but more weakly—drive receiving neurons in the transcallosal zone [3, 9, 35, 36]. Similarly, lateral intrinsic connections have been reported to take over driving functions in adult plasticity ([37], see also [38] for primate work).

## 3. Morphological Similarities between Lateral Intrinsic and Callosal Connections

Intralaminar connections running horizontally provide the numerically strongest synaptic input to both excitatory [39] and inhibitory circuits [40] within the primary visual cortex. Surprisingly, a recent study claims that synapses formed by long-range projections from outside the functional columns' range clearly outnumber local synapses giving more emphasis to those connections [41]. Although being in an anatomically unique localization within the brain's circuits callosal connections share important anatomical (and functional) properties with that intracortical network of long-range lateral connections. 

Long-range lateral connections confined to the primary visual cortex of one hemisphere display a patchy reciprocal network of axon terminals extending over horizontal distances of up to 8 mm (for review [42]). 

Like their “relatives” confined to the same hemisphere [43–45], callosal terminals arborize in the target zone at more or less regular intervals of 100–2000 *μ*m [46, 47] and ca. 980 *μ*m [19] and interconnected neuron populations tend to form clusters [22, 48–51]. Those clusters have been shown to coincide with orientation domains, indicating connections between neurons of similar orientation preference [19, 51], like it has been observed for long-range lateral connections [51–54]. Interestingly, in the lateral-eyed tree shrew, the congruence between intrinsic and callosal circuits seems to be broken and callosal fibers are less specific [18]. 

Yet another topographical similarity between intrinsic and interhemispheric circuits might render visual callosal connections a true subset of long-range lateral connections. The latter exhibit elliptic axonal arbor fields interconnecting not only neurons of the same preference orientation but among them those with receptive fields aligning along their axis of colinearity within the visual field (cat: [55], tree shrew: [56], squirrel monkey: [57]). This axial selectivity was hypothesized to be the anatomical substrate for the physiological finding that responses to optimally oriented stimuli in the classical receptive field of a neuron are enhanced when collinearly aligned contours are presented outside the classical receptive field [58, 59]. Astonishingly, the same kind of anatomy was described for axon arbors of cat callosal projection neurons in the target hemisphere [19]. 

This suggests a close relation between the Gestalt criteria like common shape (orientation selectivity) and colinearity (axial selectivity) and the topology of both long-range intrinsic and callosal connections. One could imagine that both types of lateral network equally support perceptual grouping by modulating the saliency of distributed cortical responses in a context-dependent way [55] and thus would be members of the same circuit as suggested earlier [22]. 

Further, the postnatal development of callosal connections undergoes similar phases [47] as that of long-range intrinsic connections [60]. Callosal axons are initially imprecise and exuberant and attain their adult specificity by elimination of ectopic axon terminals [61]. Like for intrinsic connections [62–64], normal visual experience is necessary to eliminate these ectopic connections [48, 65–69]. Finally, both circuits exhibit a high degree of selectivity in the adult, and both projections are susceptible to experience-dependent modifications during development in the same manner [51].

## 4. Stimulus-Dependent Gain of Callosal Action

In earlier studies of visual callosal function, the corpus callosum has often been transsected and the optic chiasm split in order to separate the callosal from the geniculocortical input [3, 70]. This is a rather invasive approach as it abolishes all fibres from the nasal retina and thus the major input to central primary visual cortex. Further, in the recovery period after the split-chiasm surgery and before the actual measurement rearrangements of connectivity or synaptic strength are possible. Cooling deactivation of the visual cortex was introduced by Payne and colleagues [71] in studies of interhemispheric interactions and was later extended to other studies [72–74]. It is less invasive than sectioning or lesioning and the effects are reversible. Although deactivating the 17/18-border region does not directly interrupt callosal fibres, the anatomy of the visual pathways assures that this method is adequately suited to reveal the influence of the interhemispheric projection on the other hemisphere. 

In a series of deactivation experiments in ferrets and cats, we recorded optical images, single unit [75, 76] and local field potential data [77, 78] while presenting different stimuli covering both hemifields. We positioned a cooling device onto the previously identified 17/18 border in the contralateral hemisphere in order to reversibly deactivate callosal input from the transition zone and the adjacent central parts of areas 17 and 18. 

The results from optical recordings with continuous whole-field gratings demonstrate that maps of orientation preference in both ferret areas 17 and 18 get weaker and less specific when cooling the contralateral hemisphere, predominantly in the 17/18-border zone. As this zone receives strong callosal input [13, 79] removing this input degrades the differential responses to gratings particularly strongly in that region. However, as in cats [36], the lateral influence spreads into areas 17 and 18. This is still in agreement with the anatomy as, in particular, area 18 in the ferret is densely linked transcallosally [13]. 

At the electrophysiological level, both increases and decreases are observed with grating stimulation but deterioration of the responses predominates [75, 76]. When probing the circuit with random dot textures (RDT), a visual stimulus that activates more neurons but in a less selective manner than gratings [80, 81], the callosal influence grows larger and almost exclusively excitatory [76]. This also holds as largely true when correcting for differences in contrast and baseline spike rate. Chance et al. [82] suggested that within active cortical circuits, the overall level of synaptic (background) input to a neuron acts as a gain control signal that modulates responsiveness to an excitatory drive. This means that, naturally, amplification can act better when cortical neurons are stimulated in an unselective manner like with RDTs and when overall background levels of synaptic activity are not yet saturated like it might be the case with gratings. Our results indicate that the gain of the input delivered via callosal connections is dynamically adapted to the feedforward drive by the external stimulus via the geniculocortical loop and probably also to the lateral intrahemispheric drive. The latter must be the case as the global nature of RDTs is only revealed when considering the larger context outside the classical receptive field. Such a joint stimulus-dependent gain control by lateral intrinsic and callosal circuits could amplify small signals such as weakly tuned input delivered by geniculocortical afferents [83, 84]. 

## 5. Excitatory and Inhibitory Nature of the Callosal Circuit 

The majority of callosal projecting neurons are of excitatory nature and in both, carnivores and rodents, only a few directly projecting inhibitory neurons have been observed [85, 86]. Their target cells in the receiving hemisphere are mainly excitatory neurons [22, 46] and mainly excitatory synapses on pyramidal and spiny stellate have been reported [19, 20, 87]. However, some projections onto inhibitory neurons also exist [88]. 

Most of the long-range intrinsic axon collaterals within early visual areas also contact other pyramids and the majority of axon boutons are excitatory [89–91]. In summary, only about 5% of the postsynaptic structures of long-range intrinsic connections in cat primary visual cortex is GABAergic [89, 90]. Studies from macaque monkey indicate that long-range connections contact dendrites of spiny and nonspiny cells in the proportion to which these cell types occur in the cortex (ratio spiny: nonspiny = 80%: 20%) [91, 92]. Reconstruction of biocytin-labeled large basket cells revealed that, in cat areas 17 and 18, the density of inhibitory boutons is highest close to the core of the injection site and the longer-range collaterals are slightly less selective for isoorientation domains than excitatory connections [93, 94]. In summary, the inhibitory network extends more locally than the excitatory network, and it is less selective yet involves more neurons [94, 95].

If we extrapolate from there to the network linking the two hemispheres, we would expect both inhibition and excitation with a strong bias towards the excitatory influence mediated by visual callosal connections. We would also expect some stimulus dependency in both excitatory and inhibitory actions. This is indeed what can be observed. 

In the spiking activity of both ferret and cat primary visual cortex, we find more facilitating than suppressive actions of callosal input [75, 76]. This is roughly in line with previous experiments applying either cooling deactivation [71, 96] or GABA/bicuculline infusion [97] to the contralateral hemisphere in cats. Payne et al. had reported a more balanced picture of inhibitory and excitatory interactions and a layer dependency. 

In our most recent study, we compared different visual stimuli and observe a stimulus dependency in the balance of excitation and inhibition contributed via the interhemispheric connection [76]. With high contrast full-field gratings, about 7% of all actions exhibit significant inhibitory character, whereas 48% are significantly excitatory. With a lesser salient and unstructured stimulus—random dot textures (RDT)- more cells are significantly affected (73% as opposed to 58% with gratings) and almost exclusively in a facilitating manner. In order to exclude that the larger callosal action was due to unselective recruitment of a larger population of neurons with RDT, we increased the orientation content by elongating the dots to form randomly scattered bars of a certain orientation. Responses to this control stimulus are also more strongly and exclusively excitatory biased by callosal input. This led us to conclude that strength and nature of callosal actions onto their target cells in primary visual cortex are not easily related to the presence or absence of the orientation component. Rather, the balance between excitation and inhibition depends on the local and global composition of the external stimulus driving the system. An oriented grating, which selectively recruits interconnected populations of similar orientation preference all over, not only provokes more recurrent isoorientation excitation than RDT but also is likely to be balanced by recurrent inhibition [98]. The latter can be mediated by inhibitory neurons from the contralateral hemisphere. However, in accordance with the anatomical ratios of long-range excitatory and inhibitory long-range circuits facilitating influences on spiking activity dominate and were never outnumbered by suppressive actions (between 10–30%). Individual suppressive actions can be prominent but are only unravelled with adequate stimulation [75, 76].

With whole-field gratings more inhibitory effects are observed in local field potentials indicating that not all possible transcallosal inhibition becomes suprathreshold [78].

## 6. Callosal Input and Response Selectivity

In Schmidt et al. [75], it has been shown that the subpopulation of neurons preferring cardinal contours are more affected than others by callosal input in their responsiveness. This can be also observed for the population spiking data in cats ([76] not shown). A similar dominance for neurons preferring cardinal contours is observed for stimulus-evoked synchronization [77]. 

Higher numbers of neurons preferring contours of cardinal orientations have been counted in the central visual field of both cats [48, 99, 100] and macaque monkeys [101]. In accordance with this, a larger cortical area is devoted to the representation of cardinal orientations in both ferrets ([102], our own baseline data in [75]) and cats [103] and responses are usually more vigorous and apparently faster when recorded with intrinsic signal imaging [104]. 

Exactly those neurons are more susceptible to the lack of callosal input than neurons preferring oblique contours indicating an asymmetry in the underlying network facilitating cardinal responses. Such asymmetries have not been observed for long-range intrinsic connections but are not excluded since this question was never specifically addressed by previous anatomical studies.

However, true changes in orientation and direction indices constitute only a small fraction of the total selectivity in deactivation studies [75, 76]. If at all, direction selectivity changes are usually larger than changes in orientation selectivity [76] as might be expected in particular for neurons preferring directions of motion crossing the vertical midline. 

Although isooriented stimuli with opposite directions of motion in the two hemifields have not been compared directly with those for coherent motion, the results of previous deactivation studies (S3 stimulus in [77, 78]) support the notion that among neurons with the same orientation preference those preferring similar directions of motion are preferentially linked via the corpus callosum as suggested before [105]. Along a similar line of evidence, asymmetric callosal influences on the two directions of motion have been reported early [71] and neurons preferring horizontal motion (and vertical contours) were differently affected by callosal input than others [75, 106].

This has been recently explicitly tested using Gabor stimuli centred on receptive fields close to the vertical meridian representation (Peiker, Schmitt, Wunderle, Eriksson, Schmidt, unpublished observation). When the direction of movement of a vertically oriented grating patch matched the movement out of the cooled hemifield responses were selectively and more strongly impaired than those to the opposite direction into the cooled hemifield. 

Interestingly, Girardin and Martin [107] attribute effective changes in preferred orientation of area 17 single cells observed with local GABA application but not with local cooling [108]. These relatively small changes might have escaped other investigations of lateral including callosal input because of the undersampling of orientations. Usually no differences smaller than 22.5° are tested. 

However, it might also be possible that connections probed in the area 17 deactivation study rather belong to the local short-range network as the distance between GABA infusion and recording sites was smaller than 500 *μ*m [107]. Here, the circuit is rather unselective and the inhibitory network might be much stronger than over larger distances ([94], for review [42]).

When deactivating local connections between columns of orthogonal orientation in area 18, broadening of orientation tuning curves has been observed in 65% of the neurons [109]. This number decays to 5% when deactivating sites of similar preference [110], which is more comparable to the situation when deactivating long-range callosal input. As Girardin and Martin [107] we also do not note large absolute changes in bandwidth ([75, 76] (data not shown)). 

## 7. Callosal Input and Multiplicative Scaling

In general, despite significant response changes, neurons largely keep their tuning profiles in the absence of lateral input. Wunderle et al. [76] approached the modifications of the tuning curves by a linear model and concluded that, in the majority of cases, they can be well described by the multiplication of the tuning curve with a constant factor. The bigger the rate decreases when lacking callosal input the more does the modification approximates a multiplication of input rates. Much fewer units exhibit a pronounced additive shift. They are mainly observed with grating stimulation and then are often accompanied by rate increase during cooling deactivation. This points towards a positive correlation between additive scaling and inhibition on the one hand, and multiplicative scaling and excitation on the other [76]. It also emphasizes that the transition between the two types of action is continuous and—as the excitatory-inhibitory balance—input dependent. Multiplicative scaling mainly preserves the neuron's response selectivity. It seems to be a dominating mechanism of action in corticocortical circuits as it can be deduced—even though not explicitly stated—from publications about feedback [111], contextual modulation [112], and spatial attention shifts [113, 114]. 

## 8. Callosal Interactions and Timing

Isooriented neurons in both hemispheres can synchronize their activity, a function that is interrupted when sectioning the corpus callosum in cats [115] and monkeys [116]. When gratings of the same orientation are presented simultaneously to both hemifields, the coherence can increase both between the hemispheres in both ferrets and humans [117–121] and within one and the same receiving hemisphere [77, 78]. 

Influences on precise local and interhemispheric timing of responses are in accordance with the anatomy of callosal axons. In general, the majority of callosal projection fibres reveal short latencies between the two hemispheres of about 2–6 ms [21]. This is enabled by relatively fast conduction delays [122]. A diversity of callosal axon diameters [5, 14–126] corresponds in turn to different axonal conduction velocities, which compensate for offsets in distance [127]. In agreement, computer simulations have shown that despite a large divergence and clustering of axon terminals [8], spikes can arrive synchronously at the target synapses [122]. Because these targets are preferentially neurons of similar orientation preference as the projection neurons on the other side, precise temporal interactions between the hemispheres are expected to be stimulus dependent.

In accordance, local synchronization and desynchronization of multiple sites close to the 17/18 border of the receiving hemisphere have been shown to be mediated by isooriented but not by cross-oriented input from the contralateral hemisphere [77]. Similarly, synchronization between the hemispheres occurs more likely for collinear and like stimulation [117, 118].

Significant effects of callosal input on the synchronization behaviour in the receiving hemisphere cannot be observed when the two hemifields are stimulated unequally, that is, with two gratings of orthogonal orientations or moving into opposite directions. Here, different neuronal populations are stimulated which are probably not selectively interconnected via the corpus callosum—neither by the excitatory nor the inhibitory projection. Therefore, a deactivation of one of the two pools will not have a significant influence on the timing within the other population. 

In the absence of patterned stimulation (isoluminant monitor) deactivating the contralateral hemisphere increases the overall synchronization between various sites within the receiving hemisphere [77] and decreases the spiking variability [128]. This might indicate that because of their target selectivity callosal connections provide correlated input and thus also correlated noise to the target neurons [129], which increases variability and thus decreases synchronized firing between distant sites [130].

## 9. Callosal Input and Binocularity

In normal cats, callosal connections as intrinsic connections [131] do not seem to necessarily interconnect domains of the same eye with each other [14, 15, 51]. Rather, ocular specificity of connections seems to depend on the retinotopic position; for example, neurons in the two hemispheres receiving input from the same retina part are selectively linked [14, 15]. This implies connections between neurons driven by the same eye as well as between neurons driven by different eyes. 

Earlier experiments sectioning the corpus callosum or lesioning the contralateral cortex in cats claimed that callosal afferents contribute a major part to the binocularity of callosal neurons in cats [132–135]. However, this result was not confirmed by other studies [136–138] and a developmental study indicated that there is a postnatal critical period for the influence of corpus callosum section on binocularity [139]. 

In small rodents, where the majority of the retinal fibres cross at the chiasm, the contribution of callosal input to binocularity and its development is inevitably larger (for review [140]). It cannot be excluded, that visual callosal connections cover a different spectrum of functions in these species than in carnivores and primates.

Our own findings using reversible deactivation in cats do not reveal any significant reduction of binocular units at the 17/18 border [106]. This strongly indicates that—in stereoscopic mammals—binocularity as such does not depend on the integrity of the callosal network but rather on the geniculocortical input as would be expected from any long-range lateral network intrinsic to one hemisphere. However, responses evoked by the two eyes separately were differently affected supporting a role of the corpus callosum in stereoscopic function as suggested before for humans [141, 142] and animals with disparity selective neurons [138, 143]. In ferrets, who also have a large proportion of disparity selective neurons [144], we observe a more complex influence of callosal input on vertically preferring units than on others. This is compatible with the interpretation that those units, which presumably participate in horizontal disparity coding at the midline, are under the control of callosal interactions [75]. However, it does not necessarily distinguish the callosal as different from the intrahemispheric circuits. The latter could serve the same function in more peripheral acallosal but binocular representations of the visual field. In agreement, these parts reveal normal stereovision in the split-brain condition in humans [142]. 

## 10. Comparison between Feedback and Interhemispheric Circuits

Methodologically, all manipulations applied to study callosal function like cutting, lesioning, or cooling inevitably disable direct or indirect lateral projections from both contralateral areas 18 and 17 or even more extrastriate cortices to the recipient transcallosal zone. 

Thus, visual callosal projections as a “broad band” interhemispheric connection could be also viewed as a special type of feedback (and even feedforward) connection. Anatomically, feedback connections from higher visual areas to primary visual cortex also exhibit anisotropically arranged clusters and thus a topographical relationship with the functional architecture of the interconnected structures as intrinsic connections do (macaque [145, 146]). They have been shown to integrate from a more comprehensive part of the visual field than the long-range intrinsic circuits and thus to be apt to contribute information from a larger modulatory surround to which intrinsic circuits do not have access to (for review [147, 148]). One of the main conclusions is that feedback connections are important for differentiating a figure from the background, particularly in the case of low salience stimuli [149].

Surprisingly, in former deactivation studies in cats, extrastriate areas like posterior middle suprasylvian cortex (pMS) [73, 74] and area 21 [111, 150] have been demonstrated to already influence basic response properties of neurons in early visual areas like orientation or direction selectivity. This was not the case when deactivating the feedforward loop from area 17 to 18 [151] and from area 17 to area 21a [152].

Earlier, we had observed that the impact of deactivating a projection area was proportional to the density of this area's projecting fibres to its target area, that is, area 18 [73, 153]. Having applied the same technique to the same animal using the same grating stimulus [76] puts us in the position to compare also the functional impact of the prominent pMS feedback circuit with that of callosal input. The former projection to central area 18 is numerically much stronger than the latter from the contralateral hemisphere (26.5% versus 4.7% of all inputs from visual structures [154]). For the callosal inactivation study [76], the strength of the callosal input might be slightly underestimated since the majority of our units stems from neurons located very close to the densely interconnected 17/18 border. 

Both feedback and interhemispheric connections do not instruct the layout of orientation preference maps but the loss of map vector strengths is indeed much stronger without pMS feedback (up to 50% [73]) than without the contralateral input (20–25% [75, 76]). 

Therefore, it is surprising that, on average, single unit spike rates to moving gratings in cat early visual areas are only slightly more affected when deactivating pMS feedback projections ([73], estimated mean response change −18%) than by removing callosal input ([76], mean response change: −14%). However, changes in direction selectivity of single units and maps related to pMS input are relevant [73, 74], whereas direction selectivity changes related to callosal input are tiny [76]. 

In summary, the comparison points out that despite topographical similarities (patchiness) both quantitative and qualitative differences exist between the functional impact of feedback and interhemispheric connections to primary visual cortex. These differences also do not easily correlate with the anatomical numerical differences confirming that the majority of interhemispheric input (as approached by the cooling technique) has different characteristics than intrahemispheric feedback circuits. 

## 11. Conclusion 

In the present paper, recent studies on the physiology of visual callosal connections of cats and ferrets are discussed. Most of these recent findings have been obtained by reversibly deactivating the contralateral visual areas, a condition that in human patients resembles a hemianopia caused by a unilateral occipital lesion. In these patients, visual processing in the intact hemifield is disturbed [154, 155]. In agreement, our animal studies reveal that contralateral deactivation exerts a considerable impact on intrahemispheric processing of visual responses. 

Namely, it is observed that the callosal influence in early visual areas—although largest close to the areal border between areas 17 and 18—continues widely into both areas. In accordance with the anatomy of the callosal circuit, actions occur preferentially between coactivated neurons of similar orientation (or direction) preference. In the majority of cases, they are excitatory, do not alter response selectivity dramatically, and can be described as a multiplicative scaling of responses. Additive tuning shifts occur less frequently and more often with gratings than with lesser salient stimuli. Similarly, different excitatory-inhibitory ratios are observed with different input regimes and thus seem to reflect a dynamical adaptation of the callosal and intrinsic circuits to the external feedforward drive via the geniculocortical loop. We hypothesize that the transition from modulatory multiplicative to additive driving action as well as the transition from excitation to inhibition is a continuum rather than a discrete step. The interhemispheric circuit cannot be decoupled from the remaining cortical network. Thus, the transition between the different callosal actions will depend critically on the actual contribution of all possible feedforward, lateral, and feedback input sources. 

Taken together anatomical and functional evidence from corticocortical and lateral circuits, we further come to the conclusion that visual callosal connections share a majority of anatomical and functional features with lateral connections on the same hierarchical level and less with feedback connections. This might justify interpreting experimental results about the callosal circuit with respect to lateral connectivity in general. 
